# Relative risk of cardiac mortality and dosimetric comparison among three-dimensional radiotherapy, volume-modulated arc therapy and proton beam in vertebral-body reduced-dose craniospinal irradiation

**DOI:** 10.1093/jrr/rraf032

**Published:** 2025-06-10

**Authors:** Chonnipa Nantavithya, Anussara Prayongrat, Sornjarod Oonsiri, Kanjana Shotelersuk

**Affiliations:** Division of Radiation Oncology, Department of Radiology, Faculty of Medicine, Chulalongkorn University, 1873, Rama 4 Rd., Patumwan, Bangkok 10330, Thailand; Division of Radiation Oncology, Department of Radiology, King Chulalongkorn Memorial Hospital, Thai Red Cross Society, 1873, Rama 4 Rd., Patumwan, Bangkok 10330, Thailand; Division of Radiation Oncology, Department of Radiology, Faculty of Medicine, Chulalongkorn University, 1873, Rama 4 Rd., Patumwan, Bangkok 10330, Thailand; Division of Radiation Oncology, Department of Radiology, King Chulalongkorn Memorial Hospital, Thai Red Cross Society, 1873, Rama 4 Rd., Patumwan, Bangkok 10330, Thailand; Division of Radiation Oncology, Department of Radiology, King Chulalongkorn Memorial Hospital, Thai Red Cross Society, 1873, Rama 4 Rd., Patumwan, Bangkok 10330, Thailand; Division of Radiation Oncology, Department of Radiology, Faculty of Medicine, Chulalongkorn University, 1873, Rama 4 Rd., Patumwan, Bangkok 10330, Thailand

**Keywords:** craniospinal irradiation, CSI, proton, VMAT, cardiac mortality, pediatric brain tumor, vertebral body reduced dose

## Abstract

We aimed to compare dose to organs at risk (OARs) and the excess relative risk (ERR) of cardiac mortality among three-dimensional conformal radiotherapy (3D-CRT), volume modulated arc therapy (VMAT) and proton beam therapy (PBT) in craniospinal irradiation (CSI). CSI plans of 3D-CRT, VMAT and PBT were generated. Vertebral body reduced dose (VBR)-CSI according to the International Society of Paediatric Oncology recommendation was used for VMAT and PBT. We delineated two sets of target volumes, i.e. target volume (TV) 1 for brain and thecal sac and TV2 for the vertebral body. Two sets of CSI dose, 23.4 and 36 Gy, were prescribed for TV1, and VBR doses, 18.4 and 20 Gy, were prescribed for TV2. For 3D-CRT, we prescribed a dose only to cover TV1. For VMAT and PBT, 23.4/18.4Gy and 36/20 Gy in 13 and 20 fractions were optimized. To evaluate the ERR of cardiac mortality compared with the normal population, we incorporate the mean heart dose with the linear model. In a total of eight patients, 48 treatment plans were generated (24 plans for each dose set). PBT showed the lowest mean dose to all OARs, i.e. heart, lung, liver, kidney, esophagus, oral cavity, thyroid and vertebral body. PBT showed significantly less ERR of cardiac mortality compared with 3D-CRT and VMAT for both 23.4 and 36 Gy prescriptions. With VBR-CSI, PBT reduced the mean dose to all OARs and significantly reduced the ERR of cardiac mortality compared with 3D-CRT and VMAT. The advantage of PBT was manifest, especially with high-dose CSI.

## INTRODUCTION

Craniospinal irradiation (CSI) is one of the treatment modalities for pediatric brain tumor, including embryonal tumor, non-germinomatous germ cell tumor and other CNS tumors with spinal seeding. While it improves survival outcomes, CSI-related toxicities remain a major concern that can lead to long-term morbidities and diminish quality of life [1]. For decades, photon or X-ray radiation therapy (XRT) has been the standard treatment for CSI. Several radiation techniques have been developed to improve outcomes. Although advanced XRT techniques such as intensity-modulated radiotherapy (IMRT) and volumetric arc therapy (VMAT) can reduce the high doses of radiation to normal organs and provide better conformity to the target volume, they expose larger volumes of normal tissue to lower doses of radiation compared with three-dimensional conformal radiotherapy (3D-CRT) [2–4]. Recently, proton beam therapy (PBT) has been adopted for the treatment of pediatric tumors. Due to its Bragg peak characteristics, PBT delivers almost no exit dose, reducing radiation exposure to adjacent normal organs [5, 6]. Previous studies have shown acceptable oncological outcomes with PBT CSI (P-CSI), similar to those with photon beam CSI (X-CSI) [1, 7–13]. In addition, P-CSI offers a lower risk of late toxicities, including certain hormonal deficiencies and the need for hormone replacement therapy [8, 13]. For second cancers, although not statistically significant, the incidence rate seemed to be lower in the P-CSI group [7]. Importantly, the dose from CSI could expose to heart. Data from survivors of pediatric cancers have shown a relationship between radiation dose to the heart and late cardiac mortality in children who received radiation [14]. Since there are limited clinical data on the rate of heart disease following CSI, predictive models incorporating the mean heart dose to assess the excess relative risk (ERR) of cardiac mortality have been introduced [15, 16].

Not only late toxicities affecting pediatric brain tumor patients; hematologic toxicity, particularly neutropenia, is also one of the most common acute toxicities that can interrupt the radiation course or require intervention [17, 18]. For 3D-CRT, even though the vertebral body is not the target volume, the exit dose is inevitably exposed to nearly the entire vertebral body, which is composed of spinal marrow. In contrast, we could spare the vertebral body from the radiation dose with P-CSI, thereby reducing hematologic toxicity and other acute toxicities, such as nausea, vomiting, dysphagia and oral mucositis. Previous studies have reported that acute toxicities from P-CSI are acceptable and less frequent than those from historical X-CSI [12, 13, 19–22]. For IMRT or VMAT, vertebral body-sparing CSI is feasible. However, scattered radiation dose to adjacent organs remains unavoidable.

For vertebral sparing CSI, the steep dose gradient at the anterior and posterior parts of the vertebral body may result in spinal abnormalities, particularly in the immature skeleton [23]. Previous studies have shown evidence of scoliosis in neuroblastoma and Wilms’ tumor patients treated with CSI who did not receive whole vertebral body radiation [24, 25]. As a result, the recommendation is to include the vertebral bodies at the same level as the target volume in the treatment field. Recently, the European Society for Paediatric Oncology (SIOP) working group has reported the consensus recommendations on vertebral radiotherapy dose for CSI [26]. To minimize acute toxicities while preventing skeletal abnormalities, they suggested including the vertebral body in the target volume but delivering a lower dose than to the thecal sac. However, both dosimetric comparison studies and clinical research on the outcomes of vertebral-body reduced-dose (VBR)-CSI remain limited.

Therefore, we conducted a dosimetric study to compare organs-at-risk (OAR) doses in CSI among three techniques, 3D-CRT, VMAT and PBT. VBR-CSI was applied in advanced techniques, VMAT and PBT, in accordance with the SIOP recommendations. As mentioned, clinical data on cardiotoxicity from CSI remain limited, especially for PBT. To address this, we also aimed to estimate the ERR of cardiac mortality, compared to the general population. This was achieved by incorporating the mean heart dose from each technique into a linear risk model, following the latest SIOP-recommended methods for vertebral body irradiation in both VMAT and PBT [14, 15].

## MATERIALS AND METHODS

**Fig. 1:**
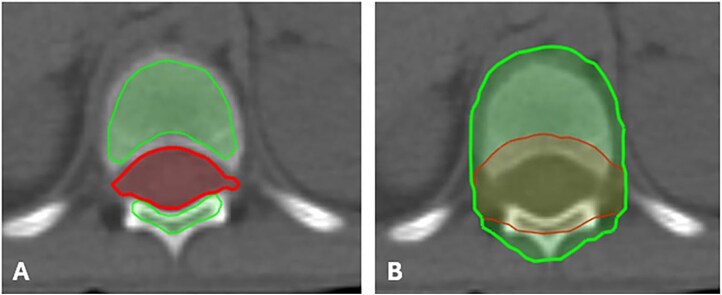
CTV1 (red) and CTV2 (green) (A), PTV 1 (red) and PTV 2 (green) (B).

Pediatric cancer patients under the age of 15 who had previously received X-CSI at King Chulalongkorn Memorial Hospital in 2020 were retrospectively reviewed. Their CT simulation images were used to re-delineate the target volume (TV), including the clinical target volume (CTV) and planning target volume (PTV), with the PTV expanded by 0.5 cm in all directions from the CTV, along with OARs. For PBT, CTV was used for robust optimization. For X-CSI, i.e. 3D-CRT and VMAT, both PTV and CTV were used for treatment planning and optimization. The definition for TV1 was the target volumes of the entire brain and thecal sac, while the TV2 definition was the target volumes of the vertebral body and arc (Fig. 1). In each radiation technique of each patient, we prescribed two common CSI doses, i.e. 23.4 and 36 Gy. For 3D-CRT, we prescribed a dose to only TV1. In contrast, for VMAT and PBT, we prescribed a dose to both TV1 and TV2 using the simultaneous integrated boost (SIB) technique. The dose for TV1 and TV2 will be 23.4 and 18.4 Gy, respectively, and 1.8 and 14.15 Gy per fraction, respectively, for the prescribed dose set of 23.4 Gy. For the dose set of 36 Gy, the TV1 and TV2 will be 36 and 20 Gy, respectively, 1.8 and 1.25 Gy per fraction.

**Fig. 2:**
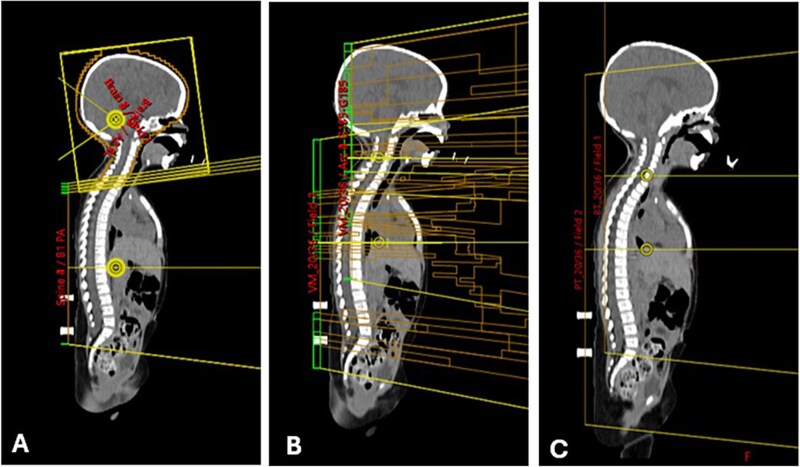
Treatment fields of 3D-CRT (A), VMAT (B) and PBT (C).

**Fig. 3:**
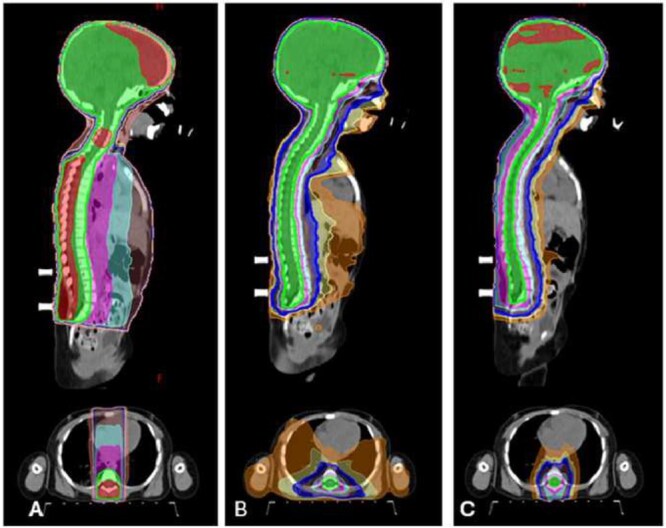
The dose distribution of 3D-CRT (A), VMAT (B) and PBT (C). Red line = 40 Gy, Green line = 36 Gy, Purple line = 30 Gy, Cyan line = 25 Gy, Brown line = 20 Gy, Blue line = 15 Gy, Yellow line = 10 Gy, Orange line = 5 Gy.

For the treatment plan, the 3D-CRT technique utilized two lateral oblique fields for cranial irradiation, including the upper cervical spine, with 6 MV photon energy. Below the upper cervical spine, extending to the end of the thecal sac, posterior–anterior (PA) fields were used with 6–10 MV energy, depending on the patient’s size. The gantry and collimators of the lateral oblique fields were tilted to prevent beam intersection from the divergent angles. To minimize cold and hot spots at the field junctions, the feathering technique was applied. For both VMAT and PBT, treatment plans were generated based on prescribed dose constraints that were adopted and modified from previous literature and applied in our institution (Supplementary Table S1) [27, 28]. These VMAT plans were designed using 6 MV photon beams, with three full arcs in the head region and two arcs in the spine region, employing two or three isocenters. For PBT, we employed intensity-modulated proton therapy with robust optimization using pencil beam scanning. This technique accounted for a setup uncertainty of 5 mm and a range uncertainty of 3.5%. Each plan was optimized with priority given to ensuring adequate dose coverage of the target volume, followed by meeting the specified dose constraints of OARs. Contouring, treatment planning and optimization were performed using the Varian Eclipse Treatment Planning System, version 16.1. Treatment field and dose distributions for each technique are shown in Figs 2 and 3, respectively.

A total of six treatment plans were created for each patient: 3D-CRT, VMAT and PBT for both the 23.4 and 36 Gy prescription doses. The dose to OARs was recorded. The homogeneity index (HI) of the vertebral body was analyzed using the low dose to the vertebral body (D98, minimum dose received by 98% of the vertebral body), the high dose to the vertebral body (D2, dose to the hottest 2% of the vertebral body), and the prescription dose, derived from Wu *et al*. [29] as shown in the formula below.


$$ \mathrm{HI}=\left(\mathrm{D}2\hbox{--} \mathrm{D}98\right)\!/ \mathrm{Prescription}\ \mathrm{dose}\ \times\ 100 $$

A pilot study was conducted, using data from three cases, and the appropriate sample size was calculated based on the mean heart dose. A paired *t*-test was used to compare 3D-CRT vs VMAT, 3D-CRT vs PBT and VMAT vs PBT for both the 23.4 and 36 Gy prescription doses.

To evaluate the ERR of cardiac mortality compared to the normal population, we incorporated the mean heart dose for each prescription dose into a linear model derived from Tukenova *et al*. [14], as shown below.


$$ \mathrm{ERR}=\exp\ \left[1+\mathrm{\alpha}\ \mathrm{dose}\right] $$

$$ \mathrm{\alpha} =0.6. $$

The differences in radiation dose and the ERR of cardiac mortality among three techniques were analyzed using repeated analysis of variance (ANOVA) for each prescribed dose. Pairwise comparisons were performed using the *t*-test for normally distributed data (i.e. 3D-CRT vs VMAT, 3D-CRT vs PBT and VMAT vs PBT). For non-normally distributed data, the Friedman test and Wilcoxon signed-rank test were used for analysis. Additionally, patients were divided into two age groups, <10 years and ≥10 years. The dose to OARs and the ERR of cardiac mortality were also analyzed according to these age groups. A *P*-value of <0.05 was considered statistically significant.

## RESULTS

Based on the pilot study, the recommended sample size was three patients. However, to allow evaluation across each age subgroup, we included all eight patients who received X-CSI in 2020, resulting in 48 treatment plans: 24 plans for the 23.4 Gy prescription (3 plans each of 3D-CRT, VMAT and PBT for each patient) and 24 plans for the 36 Gy prescription (3 plans each of 3D-CRT, VMAT and PBT for each patient). Patients’ characteristics are shown in Supplementary table S2. PBT showed the lowest dose to all OARs, with statistical significance in almost all organs compared to the other two techniques. Additionally, VMAT showed statistically lower doses to almost all organs compared with 3D-CRT, except for the liver, kidney, oral cavity, mean lung dose and lung V5%. There was no significant difference in the HI for the 23.4 Gy prescription. However, for the 36 Gy prescription, HI of both VMAT and PBT were significantly better than 3D-CRT (Tables 1 and 2). In the subset analysis based on age groups, there were four cases in the younger age group (<10 years) and four cases in the older age group (≥10 years), and the results were mostly consistent with those of the overall population (Supplementary Tables S2 and S3). Regarding the ERR of cardiac mortality, PBT demonstrated a significantly lower ERR compared with both 3D-CRT and VMAT in both prescriptions. In subgroup analysis by age group, the ERR of cardiac mortality was again lowest for PBT, followed by VMAT and 3D-CRT (Table 3).

**Table 1:** Dosimetric comparison among radiation techniques for the prescription dose of 23.4 Gy

**Organ**	**3D-CRT Gy (SD)**	**VMAT Gy (SD)**	**PBT Gy (SD)**	***P*-value**
Mean heart dose, Gy	12.15 (1.62)	3.99 (0.25)	0.9 (0.44)	<0.001^a^
Mean lung dose, Gy	3.64 (1.11)	6.59 (0.36)	1.95 (1.28)	<0.001^a^
Lung V20% (IQR)	3.6 (2.8–6.3)^* ¥^	0.04 (0.02–0.08)^*^	0.22 (0–1)^¥^	0.05
Lung V5%	14.76 (6.27)^*^	63.12 (5.6)^*, ¥^	12.24 (8.8)^¥^	<0.001
Mean esophagus dose	21.59 (0.9)	14.57 (0.38)	11.66 (2.1)^¥^	<0.001^a^
Dmax Esophagus, Gy	24.05 (0.86)	22.09 (1.16)	20.39 (1.35)	<0.001^a^
Mean liver dose, Gy	5.23 (0.82)	5.96 (0.72)	0.44 (0.25)	<0.001^a^
Mean kidney dose, Gy	2.83 (1.15)^*^	5.8 (1)^*,¥^	2.18 (2.09)^¥^	<0.001
Mean oral cavity dose, Gy	3.39 (1.64)^*^	7.65 (1.04)^*, ¥^	2.16 (2.44)^¥^	0.002
Mean thyroid dose, Gy	17.19 (4.68)	14.06 (1.76)	7.82 (3.28)	<0.001^a^
Mean vertebral body dose, Gy	24.44 (0.56)	22.59 (0.56)	21.11 (0.73)	<0.001^a^
D2 vertebral body dose, Gy	26.98 (0.95)	25.14 (0.52)	24.01 (0.59)	<0.001^a^
D98 vertebral body dose, Gy	22.25 (0.93)^* ¥^	19.74 (0.43)^*^	19.26 (0.28)^¥^	<0.001
HI vertebral body dose	20 (7)	23 (2)	20 (2)	0.296

Abbreviations: CRT = conformal radiotherapy, VMAT = volumetric arc therapy, PBT = proton beam therapy, SD = standard deviation, IQR = interquartile range, Vx = volume that received x dose, Dx = dose received by x% of the volume, Dmax = maximum dose, Dmin = minimum dose; HI = homogeneity index.

*, ¥, € = pairwise were statistical significantly (*P* < 0.05).

^a^ *P-*value from repeated ANOVA and all pairwise was statistically significant (*P* < 0.01).

**Table 2:** Dosimetric comparison among radiation techniques for prescription dose of 36 Gy

**Organ**	**3D-CRT Gy (SD)**	**VMAT Gy (SD)**	**PBT Gy (SD)**	***P*-value**
Mean heart dose, Gy	18.6 (2.5)^*,¥^	5.7 (0.6)^*^	1.2 (0.4)^¥^	<0.001
Mean lung dose, Gy All	5.6 (1.73)^*^	9.06 (0.65)^*,¥^	2.6 (1.64)^¥^	<0.001
Lung V20%	8.53 (4.3)	2.85 (1.62)	3.54 (3.0)	<0.001^a^
Lung V5%	22.29 (7.51)	92.01 (4.67)	14.16 (9.66)	<0.001^a^
Mean esophagus dose	33.12 (1.23)	17.22 (0.66)	14.28 (3.58)	<0.001^a^
Dmax Esophagus, Gy	36.77 (1.38)^*, ¥^	29.99 (4.26)^*^	26.63 (3.55)^¥^	<0.001
Mean liver dose, Gy	7.07 (2.69)^*^	8.19 (1)^¥^	0.52 (0.26)^*. ¥^	<0.001
Mean kidney dose, Gy	4.33 (1.77)^*^	7.17 (0.61)^*, ¥^	2.47 (2.04)^¥^	<0.001
Mean oral cavity dose, Gy	4.76 (1.75)	10.63 (1.4)	2.64 (3.26)	<0.001^a^
Mean thyroid dose, Gy	25.28 (6.38)	18.08 (3.07)	10.41 (5.05)	<0.001^a^
Mean vertebral body dose, Gy	37.44 (0.82)	31.76 (1.6)	28.1 (3.66)	<0.001^a^
D2 vertebral body dose, Gy	40.86 (1.26)	38.8525 (0.47)	36.63 (1.45)	<0.001^a^
D98 vertebral body dose, Gy	34.08 (1.38)^*, ¥^	23.13 (1.54)^*^	22.27 (2.89)^¥^	<0.001
HI vertebral body dose	19 (6)^*, ¥^	44 (5)^*^	40 (7)^¥^	<0.001

Abbreviations: CRT = conformal radiotherapy, VMAT = volumetric arc therapy, PBT = proton beam therapy, SD = standard deviation, IQR = interquartile range, Vx = volume that received x dose, Dx = dose received by x% of the volume, Dmax = maximum dose, Dmin = minimum dose, HI = homogeneity index

*, ¥,€ = pairwise were statistically significant (*P* < 0.05).

^a^ *P-*value from repeated ANOVA and all pairwise was statistically significant (*P* < 0.01).

**Table 3:** Comparison of the excess relative risk of cardiac mortality among radiation techniques

	**3D-CRT (SD)**	**VMAT (SD)**	**PBT (SD)**	***P*-value**
23.4 Gy prescription – All – <10 years old – ≥10 years old	8.29 (9.7) 9.03 (0.73) 7.56 (0.5)	3.40 (0.15) 3.36 (0.21) 3.43 (0.07)	1.54 (0.26) 1.57 (0.37) 1.50 (0.15)	<0.001^a^ <0.001^a^ <0.001^a^
36 Gy prescription – All – <10 years old – ≥10 years old	12.19 (1.51) 13.35 (1.13) 11.0 (0.68)	4.40 (0.33) 4.31 (0.47) 4.49 (0.11)	1.74 (0.25) 1.94 (0.17) 1.54 (0.11)	<0.001^a^ <0.001^a^ <0.001^a^

Abbreviations: CRT = conformal radiotherapy, VMAT = volumetric arc therapy, PBT = proton beam therapy, SD = standard deviation.

^a^ *P-*value from repeated ANOVA and all pairwise was statistically significant (*P* < 0.01).

## DISCUSSION

In the present study, we compared the dose to OARs among three common radiation techniques for CSI, following the SIOP recommendation for dose reduction to the vertebral body. Although the dose constraints for VMAT and PBT may appear different, we followed the principle of optimizing the dose to OARs to be as low as reasonably achievable. It is well recognized that proton therapy generally delivers lower doses to OARs compared to VMAT. Therefore, based on published dosimetric studies [27, 28], which we have adopted and adapted into our clinical practice, we set the dose constraints accordingly, believing they best represent real-world practice. As expected, PBT delivered significantly lower doses to OARs than both 3D-CRT and VMAT in almost all organs, with non-significantly lower doses for lung V5%, mean kidney and mean oral cavity at the 23.4 Gy prescription, and for MHD, MLD, Dmax esophagus and mean kidney dose at the 36 Gy prescription. Additionally, the mean dose with VMAT was lower than with 3D-CRT for several organs, including the heart, thyroid, esophagus and vertebral body. However, for organs not directly within the 3D-CRT treatment fields, such as the liver, kidneys, oral cavity and lungs, the mean dose with VMAT was higher than with 3D-CRT. This was due to the beam direction in 3D-CRT, which utilized opposed lateral oblique fields from the cranial to the upper cervical spine to avoid dose to the oral cavity, and a PA field directed downward, which helped reduce radiation exposure to the lung, kidney and liver. As anticipated, while VMAT reduced the high dose to several organs compared to 3D-CRT, it inevitably resulted in increased low-dose exposure when compared to both 3D-CRT and PBT. This disadvantage of VMAT, particularly the increased low-dose exposure, could contribute to a higher risk of second cancers in survivors [30]. Despite the use of a VBR-CSI technique, our findings remain consistent with previous studies, whether using passive scattering or pencil beam scanning proton therapy [6, 16, 31–35]. In the studies by Yoon *et al*. and Mu *et al*., the Dmean of the thyroid and esophagus was lowest with PBT, followed by inverse-planning XRT and 3D-CRT. For the liver, kidneys and lungs, PBT still showed the lowest mean dose, but the Dmean of inverse-planning XRT was higher than 3D-CRT in these organs, which is consistent with our results. Based on the available evidence, PBT is recommended for curative intent in pediatric cancers [36]. However, due to limited proton center availability, X-ray therapy remains an optimal option. When deciding between 3D-CRT and VMAT, the risks and benefits of each technique should be carefully considered.

From our dosimetric comparison, we anticipated the least toxicity in the PBT group, given the lower doses to OARs. However, there is no absolute threshold dose for each potential side effect. One of the most concerning late toxicities is heart disease. In adults, data from breast cancer patients have shown a linear correlation between the increasing rates of major coronary events and MHD, with no threshold, at a rate of 7.4% per 1 Gy increase in dose [37]. Recently, the Pediatric Normal Tissue Effects in the Clinic study reported hazard ratios for coronary artery disease, heart failure (HF), valvular heart disease (VHD) and any cardiac disease as 2.01, 1.87, 1.88 and 1.88, respectively, for each 10 Gy increase in MHD [38]. In children, due to the limited number of patients, the incidence rates of heart disease following CSI are less frequently reported. However, one of the largest cohorts, which included 14 358 adult survivors of childhood cancer, showed significantly increased risks of congestive heart failure (CHF), myocardial infarction (MI), pericardial disease (PD) and valvular heart disease (VHD) compared with their siblings [39]. Specifically, among 1876 brain cancer survivors, the risks of coronary heart disease (CHD), MI and PD were significantly elevated. In all cancer types, an MHD exceeding 15 Gy significantly correlated with increased risk of CHF, MI, PD and VHD. Another cohort of 4122 survivors of childhood cancer found that cardiovascular mortality risk increased significantly with MHD >5 Gy, and even more with MHD above 15 Gy, with risk ratios of 12.5 and 25.1, respectively [14]. Despite the correlation between MHD and cardiotoxicity, we still cannot specify the exact risk for patients with different MHD, as data on this remain limited. Nowadays, several radiation techniques are available for CSI, and our results showed statistically significant differences in MHD among these techniques. However, differences in MHD alone may not fully predict the risk of developing heart disease, and there are still no clinical data comparing cardiotoxicity between PBT and XRT. Since predicting the risk of cardiotoxicity in individual patients may be essential and could provide crucial information for selecting the most appropriate radiation technique, we calculated the ERR of cardiac mortality for each CSI technique in our study using a linear equation for the dose–effect relationship. Our findings support the potential benefits of proton therapy. From our dosimetric results, MHD exceeded 10 Gy in both the 23.4 and 36 Gy prescriptions of the 3D-CRT group, 3–6 Gy for VMAT and just around 1 Gy for proton therapy. When incorporating the MHD values and the linear model from Tukenova *et al*., the ERR of cardiac mortality was significantly lowest in the proton therapy group, followed by VMAT and 3D-CRT. Notably, in the younger age group and with higher prescription doses, proton therapy demonstrated a more pronounced benefit in terms of reducing cardiac mortality. In conjunction with age-specific heart disease mortality rates in the general population, this can serve as a preliminary estimate to help the medical team choose the most suitable CSI technique for each patient.

Despite the benefits of advanced techniques, including PBT, the appropriate dose to the vertebral body in CSI for pediatric patients with immature skeletal growth remains controversial [40]. Due to the steep dose gradient, the dose difference between the anterior and posterior parts of the vertebral body is much greater than with conventional 3D-CRT. Scoliosis and kyphosis have been reported in pediatric neuroblastoma patients who received asymmetrical radiation doses to the vertebral body with the risk rising significantly when the dose exceeds 17.5 Gy [25, 41]. Studies on Wilms’ tumor have also shown that doses as low as 8–14 Gy can affect the growth of the epiphysis [42]. Consequently, contouring guidelines recommend delivering at least 18 Gy to the vertebral body in neuroblastoma patients and using the same dose as the target volume for Wilms’ tumor. For CSI in pediatric brain tumors, the common prescription doses range from 23.4 to 36 Gy. With the conventional 3D-CRT technique, there is a gradual dose fall-off from the posterior spine field, inevitably delivering a high dose to the vertebral body. In contrast, advanced techniques like VMAT and proton therapy can generate a much faster dose fall-off beyond the target volume, which could lead to growth abnormalities in the vertebral body. In response to this challenge, many proton centers have included the entire vertebral body in the target volume and prescribed the same dose as the thecal sac [31, 32]. However, this method results in unnecessary high doses to organs adjacent to the vertebral body, such as the oral cavity, esophagus, heart and kidneys. A previous study found that proton therapy resulted in higher esophageal dose compared with IMRT and 3D-CRT [32]. Also, bone marrow could be depleted, resulting in anemia, leukopenia and thrombocytopenia during radiation treatment. Moreover, this could lead to shortened height. To mitigate these risks, some centers have opted for vertebral body-sparing CSI techniques [22]. A previous study reported diminished growth of the posterior part of the vertebral bodies in all cases that received vertebral body-sparing proton CSI. Furthermore, two out of five survivors in this cohort were clinically diagnosed with scoliosis. To strike a balance between minimizing the risk of vertebral body abnormalities and reducing dose to OARs, our center adopted the SIOP recommendations for vertebral body radiotherapy doses for both PBT and VMAT [26]. These guidelines recommend an anterior–posterior dose gradient of at least 5 Gy for prescription doses not exceeding 25 Gy and a dose of at least 20 Gy to the vertebral body when the thecal sac receives a dose higher than 25 Gy. For the common CSI prescription doses of 23.4 and 36 Gy, we chose the lowest recommended dose to the vertebral body: 18.4 and 20 Gy, respectively. Using this approach, unlike previous studies that prescribed the same dose to both the vertebral body and the thecal sac, our results show that the Dmax of the esophagus and the Dmean of the vertebral body were lowest in the proton therapy group, followed by VMAT and 3D-CRT.

In spite of the insights gained from our study on VBR-CSI, there are some limitations. First, as this is a dosimetric study, we cannot determine whether the significant differences observed will translate into clinically meaningful outcomes. Second, the ERR of cardiac mortality could be influenced by other factors, such as chemotherapy or the patient’s overall health status. For this reason, we did not report estimated incidence rates but instead focused on the predicted ERR based on the MHD calculated from the predictive model. However, our primary aim was to compare the ERR across the three radiation techniques, assuming that all other patient-related factors remain constant, to help guide the selection of an appropriate radiation technique. Nevertheless, if an actual incidence rate is needed, the ERR can also be applied by combining it with age-specific cardiac mortality rates in the general population. Third, our analysis only includes the dose–volume histogram data for CSI, excluding the tumor bed boost. Due to the limited number of pediatric cases, our study encompassed patients with different locations of intracranial primary tumors. This heterogeneity may introduce bias and could potentially affect the validity of comparing the boost phase of three radiation techniques. Lastly, we did not compare VBR-CSI with conventional methods, such as irradiating the entire vertebral body or completely sparing it. However, it is well established that including the entire vertebral body may unnecessarily increase radiation exposure to the bone marrow and adjacent organs, while completely sparing it can lead to complications in patients with immature skeletons. As a result, these conventional approaches are not routinely used in current clinical practice in many institutions. Meanwhile, with the increasing availability of advanced XRT and PBT, the use of an SIB has become increasingly feasible in many centers. Therefore, we believe that our comparison of VBR-CSI using VMAT and PBT accurately reflects current trends in real-world clinical practice.

## CONCLUSION

With VBR-CSI in a common prescription dose, PBT significantly reduced the mean dose to all OARs and lowered the ERR of cardiac mortality compared to both 3D-CRT and VMAT. The benefits of PBT were particularly evident with higher prescription doses (36 Gy) and in younger patients. Given the promising dosimetric results, further clinical studies with a larger cohort and longer follow-up are needed to assess the long-term clinical outcomes of proton VBR-CSI in specific pediatric diseases.

## Supplementary Material

revised_Supplementary_table_csi_dosimetric_clean_rraf032

## PRESENTATION AT CONFERENCE

55th Annual Congress of the International Society of Paediatric Oncology (SIOP 2023).
