# Supplementary material for: Relative risk of cardiac mortality and dosimetric comparison among three-dimensional radiotherapy, volume-modulated arc therapy and proton beam in vertebral-body reduced-dose craniospinal irradiation
Source: J Radiat Res. 2025 Jun 10;66(4):415–22. doi: 10.1093/jrr/rraf032 (PMC12283519; doi:10.1093/jrr/rraf032)
Supplement: revised_Supplementary_table_csi_dosimetric_clean_rraf032 [file revised_supplementary_table_csi_dosimetric_clean_rraf032.docx]

**Supplementary table 1: Dose constraints for optimization**

|  | VMAT 23.4 Gy | VMAT 36 Gy | PBT 23.4 Gy | PBT 36 Gy |
| --- | --- | --- | --- | --- |
| CTV thecal sac | D99% ≥ 100% PD | D99% ≥ 100% PD | D99% ≥ 100% PD | D99% ≥ 100% PD |
| Robust optimization | N/A | N/A | D95% ≥ 100% PD | D95% ≥ 100% PD |
| CTV vertebral body | D99% ≥ 100% PD | D99% ≥ 100% PD | D95% ≥ 100% PD | D95% ≥ 100% PD |
| Robust optimization | N/A | N/A | D90% ≥ 100% PD | D90% ≥ 100% PD |
| PTV thecal sac | D95% ≥ 100% PD | D95% ≥ 100% PD | N/A | N/A |
| PTV vertebral body | D95% ≥ 100% PD | D95% ≥ 100% PD | N/A | N/A |
| Mean lung dose | 700 cGy | 1050 cGy | 200 cGy | 300 cGy |
| Mean heart dose | 500 cGy | 750 cGy | 300 cGy | 450 cGy |
| Mean esophageal dose | 2000 cGy | 3000 cGy | 1200 cGy | 1800 cGy |
| Mean liver dose | 600 cGy | 900 cGy | 100 cGy | 150 cGy |
| Mean kidney dose | 600 cGy | 900 cGy | 200 cGy | 300 cGy |
| Mean oral cavity dose | 1000 cGy | 1500 cGy | 400 cGy | 600 cGy |
| Mean thyroid dose | 1500 cGy | 2250 cGy | 1000 cGy | 1500 cGy |

Abbreviation: VMAT = volumetric arc therapy, PBT = proton beam therapy, CTV = clinical target volume, PTV = planning target volume, PD = prescription dose, N/A = not available

**Supplementary table 2:** Patients’ characteristics

|  | All | < 10 years old | ≥10 years old |
| --- | --- | --- | --- |
| Sex (%) |  |  |  |
| Male | 75 | 50 | 100 |
| Female | 25 | 50 | 0 |
| Age (year) |  |  |  |
| Median (IQR) | 9.5 (7-12.75) | 6 (3.5-8.25) | 13.5 (11.5-15) |
| Mean (SD) | 9.38 (4.72) | 5.75 (3.30) | 13 (2.45) |
| Weight (kg) |  |  |  |
| Median (IQR) | 39.8 (15.21-44.38) | 14.48 (12.7-21.36) | 45.25 (43.13-49.5) |
| Mean (SD) | 33.48 (17.41) | 19.59 (12.13) | 47.38 (6.75) |
| Height (cm) |  |  |  |
| Median (IQR) | 131 (109.78-138) | 101.55 (85-122) | 141 (131.5-153.75) |
| Mean (SD) | 124.85 (28.40) | 105.45 (24.61) | 144.25 (16.5) |

Abbreviation: IQR = interquartile range, SD = standard deviation

**Supplementary table 3:** Dosimetric comparison among radiation techniques for prescription dose of 23.4 Gy in each age group

| Organ | 3D-CRT Gy (SD) | VMAT Gy (SD) | PBT Gy (SD) | p-value |
| --- | --- | --- | --- | --- |
| Mean heart dose, Gy |  |  |  |  |
| < 10 years old | 13.38 (1.22) | 3.94 (0.36) | 0.96 (0.61) | <0.001^a^ |
| ≥10 years old | 10.93 (0.77) | 4.05 (0.12) | 0.84 (0.25) | <0.001^a^ |
| Mean lung dose, Gy |  |  |  |  |
| < 10 years old | 4.36 (1.04) | 6.79 (0.38) | 2.56 (1.52) | 0.001 ^a^ |
| ≥10 years old | 2.93 (0.66) | 6.39 (0.21) | 1.34 (0.7) | <0.001^a^ |
| Lung V20 % (IQR) |  |  |  |  |
| < 10 years old | 6.3 (3.7-9.3) ^*,€^ | 0.07 (0.03-0.11) ^*^ | 0.22 (0.05-1.1) ^€^ | 0.07 |
| ≥10 years old | 2.9 (2.1-3.3) ^*,€^ | 0.03 (0.02-0.05) ^*^ | 0.2 (0-1) ^€^ | 0.53 |
| Lung V5 % |  |  |  |  |
| < 10 years old | 18.92 (5.34)^*^ | 65.82 (6.73)^*, ¥^ | 16.54 (10.76) ^¥^ | <0.001 |
| ≥10 years old | 10.6 (4.15) | 60.43 (2.94) | 7.94 (3.97) | <0.001^a^ |
| Mean esophagus dose |  |  |  |  |
| < 10 years old | 21.55 (0.8) | 14.58 (0.46) | 12.25 (2.55) | 0.01^a^ |
| ≥10 years old | 21.63 (1.11) | 14.57 (0.37) | 11.07 (1.68) | <0.001^a^ |
| Dmax Esophagus, Gy |  |  |  |  |
| < 10 years old | 23.9 (0.9)^*^ | 21.91 (1.13)^*^ | 20.98 (1.37) | 0.01 |
| ≥10 years old | 24.2 (0.91)^*,€^ | 22.28 (1.34)^*^ | 19.8 (1.21) ^€^ | <0.001 |
| Mean liver dose, Gy |  |  |  |  |
| < 10 years old | 5.77 (0.64)^*^ | 6.33 (0.75) ^¥^ | 0.58 (0.26)^*, ¥^ | <0.001 |
| ≥10 years old | 4.69 (0.61) ^*^ | 5.59 (0.53) ^¥^ | 0.3 (0.18)^*,¥^ | <0.001 |
| Mean kidney dose, Gy |  |  |  |  |
| < 10 years old | 3.53 (1.25)^*^ | 6.23 (1.36)^*,¥^ | 3.33 (2.52)^¥^ | 0.01 |
| ≥10 years old | 2.12 (0.45) | 5.36 (0.06) | 1.04 (0.57) | <0.001^a^ |
| Mean oral cavity dose, Gy |  |  |  |  |
| < 10 years old | 3.18 (1.04)^*^ | 8.05 (1.4)^*, ¥^ | 3.29 (3.14) ^¥^ | 0.06 |
| ≥10 years old | 3.59 (2.25)^*^ | 7.25 (0.35)^*, ¥^ | 1.03 (0.78) ^¥^ | 0.01 |
| Mean thyroid dose, Gy |  |  |  |  |
| < 10 years old | 15.27 (6.24) | 14.85 (1.24) | 9.77 (1.12) | <0.001^a^ |
| ≥10 years old | 19.11 (1.55) | 13.27 (1.99) | 5.86 (3.7) | <0.001^a^ |
| Mean vertebral body dose, Gy |  |  |  |  |
| < 10 years old | 24.27 (0.34) | 22.66 (0.52) | 21.44 (0.4) | 0.01^a^ |
| ≥10 years old | 24.61 (0.74) | 22.52 (0.66) | 20.79 (0.89) | <0.001^a^ |
| D2 vertebral body dose, Gy |  |  |  |  |
| < 10 years old | 26.40 (0.17) | 25.00 (0.55) | 24.16 (0.45) | <0.001^a^ |
| ≥10 years old | 27.56 (1.07) | 25.27 (0.53) | 23.85 (0.73) | <0.001^a^ |
| D98 vertebral body dose, Gy |  |  |  |  |
| < 10 years old | 22.61 (0.57) ^*,€^ | 19.86 (0.49) ^*^ | 19.36 (0.36) ^€^ | <0.001 |
| ≥10 years old | 21.90 (1.15) ^*,€^ | 19.63 (0.38) ^*^ | 19.16 (0.18) ^€^ | <0.001 |
| HI vertebral body dose |  |  |  |  |
| < 10 years old | 16 (3) ^*,€^ | 22 (3) ^*^ | 0.21 (2) ^€^ | <0.001 |
| ≥10 years old | 24 (8) | 24 (1) | 20 (3) | 0.342 |

Abbreviation: CRT= conformal radiotherapy, VMAT= volumetric arc therapy, PBT = proton beam therapy, SD= standard deviation, IQR= interquartile range, Vx= volume that received x dose, Dx = dose received by x% of the volume, Dmax = maximum dose, Dmin = minimum dose, HI = homogeneity index

a = p-value from repeated ANOVA and all pairwise was statistical significantly ( p<0.01), *, ¥,€ = pairwise were statistical significantly (p<0.05)

**Supplementary table 4**: Dosimetric comparison among radiation techniques for prescription dose of 36 Gy in each age group

| Organ | 3D-CRT Gy (SD) | VMAT Gy (SD) | PBT Gy (SD) | p-value |
| --- | --- | --- | --- | --- |
| Mean heart dose, Gy |  |  |  |  |
| < 10 years old | 20.6 (1.9) ^*,¥^ | 5.5 (0.8) ^*^ | 1.6 (0.3) ^¥^ | <0.001 |
| ≥10 years old | 16.7 (1.1) ^*,¥^ | 5.8 (0.2) ^*^ | 0.9 (0.2) ^¥^ | <0.001 |
| Mean lung dose, Gy |  |  |  |  |
| < 10 years old | 6.72 (1.63) ^*,¥^ | 9.52 (0.6) ^*^ | 3.52 (1.86) ^¥^ | 0.001 |
| ≥10 years old | 4.48 (1) ^*,¥^ | 8.61 (0.3) ^*^ | 1.68 (0.74) ^¥^ | <0.001 |
| Lung V20 % |  |  |  |  |
| < 10 years old | 11.51 (4.1) ^*,¥^ | 4.00 (1.46) ^*^ | 5.25 (3.34) ^¥^ | 0.001 |
| ≥10 years old | 5.64 (1.98) ^*,¥^ | 1.69 (0.61) ^*^ | 1.83 (1.43) ^*^ | 0.01 |
| Lung V5 %, |  |  |  |  |
| < 10 years old | 27.27 (6.06) | 93.18 (5.94) | 19.32 (11.53) | <0.001^a^ |
| ≥10 years old | 17.32 (5.37) | 90.85 (3.47) | 8.99 (3.7) | <0.001^a^ |
| Mean esophagus dose |  |  |  |  |
| < 10 years old | 33.18 (0.93) | 17.53 (0.59) | 16.03 (3.12) | 0.01 ^a^ |
| ≥10 years old | 33.05 (1.62) | 16.92 (0.65) | 12.52 (3.45) | <0.001^a^ |
| Dmax Esophagus, Gy |  |  |  |  |
| < 10 years old | 36.52 (1.42)^*^ | 30.25 (5.03) | 27.64 (2.73) ^*^ | 0.03 |
| ≥10 years old | 37.02 (1.5)^*, ¥^ | 29.74 (4.1) ^*^ | 25.63 (4.4) ^¥^ | 0.002 |
| Mean liver dose, Gy |  |  |  |  |
| < 10 years old | 6.98 (4.01) ^*^ | 8.64 (1.26) ^¥^ | 0.68 (0.24)^*, ¥^ | 0.02 |
| ≥10 years old | 7.17 (0.88)^*^ | 7.74 (0.45) ^¥^ | 0.36 (0.19)^*, ¥^ | <0.001 |
| Mean kidney dose, Gy |  |  |  |  |
| < 10 years old | 5.42 (1.93)^*^ | 7.4 (0.81)^*. ¥^ | 3.6 (2.43) ^¥^ | 0.01 |
| ≥10 years old | 3.24 (0.68)^*^ | 6.94 (0.28)^*, ¥^ | 1.34 (0.66) ^¥^ | <0.001 |
| Mean oral cavity dose, Gy |  |  |  |  |
| < 10 years old | 4.61 (1.33) | 11.6 (1.13) | 4.42 (4.01) | 0.009 ^a^ |
| ≥10 years old | 4.92 (2.31) | 9.67 (0.88) | 0.87 (0.57) | 0.01 ^a^ |
| Mean thyroid dose, Gy |  |  |  |  |
| < 10 years old | 22.73 (7.87) | 19.5 (2.32) | 13.36 (3.22) | 0.12 |
| ≥10 years old | 27.83 (3.98) | 16.65 (3.34) | 7.45 (5.08) | 0.001^a^ |
| Mean vertebral body dose, Gy |  |  |  |  |
| < 10 years old | 37.3 (0.58) | 32.55 (1.48) | 30.2 (3.37) | 0.04 |
| ≥10 years old | 37.58 (1.09) | 30.98 (1.47) | 26 (2.83) | <0.001^a^ |
| D2 vertebral body dose, Gy |  |  |  |  |
| < 10 years old | 40.21 (0.25) | 38.89 (0.39) | 37.3 (0.75) | <0.001^a^ |
| ≥10 years old | 41.51 (1.62) | 38.81 (0.59) | 35.96 (1.78) | <0.001^a^ |
| D98 vertebral body dose, Gy |  |  |  |  |
| < 10 years old | 34.78 (0.92) ^*, ¥^ | 23.83 (1.87) ^*^ | 23.69 (3.76) ^¥^ | <0.001 |
| ≥10 years old | 33.39 (1.53) | 22.44 (0.84) | 20.86 (0.17) | <0.001^a^ |
| HI vertebral body dose |  |  |  |  |
| < 10 years old | 15 (2) ^*, ¥^ | 42 (6) ^*^ | 38 (9) ^¥^ | <0.001 |
| ≥10 years old | 23 (6) ^*, ¥^ | 45 (2) ^*^ | 42 (5) ^¥^ | <0.001 |

Abbreviation: CRT= conformal radiotherapy, VMAT= volumetric arc therapy, PBT = proton beam therapy, SD= standard deviation, IQR= interquartile range, Vx= volume that received x dose, Dx = dose received by x% of the volume, Dmax = maximum dose, Dmin = minimum dose, HI = homogeneity index

a = p-value from repeated ANOVA and all pairwise was statistical significantly ( p<0.01), *, ¥,€ = pairwise were statistical significantly (p<0.05)
